# Effects of Trimethoprim on Three Previously Proposed Putative Biomarkers for OCT2/MATE‐Mediated Renal Drug‐Drug Interactions in Healthy Volunteers

**DOI:** 10.1002/cpt.70384

**Published:** 2026-07-04

**Authors:** Jana Picurová, Fabian Müller, Daniel Auge, Jörg König, Martin F. Fromm, Arne Gessner

**Affiliations:** ^1^ Institute of Experimental and Clinical Pharmacology and Toxicology Friedrich‐Alexander‐Universität Erlangen‐Nürnberg Erlangen Germany; ^2^ Boehringer Ingelheim Pharma GmbH & Co. KG Biberach an der Riss Germany; ^3^ FAU NeW – Research Center New Bioactive Compounds Friedrich‐Alexander‐Universität Erlangen‐Nürnberg Erlangen Germany

## Abstract

Regulatory agencies request the assessment of the potential of new drugs to cause transporter‐mediated drug‐drug interactions (DDI). This assessment can be improved by integrating endogenous biomarkers during drug development. Regarding the renal excretion of drugs such as metformin, the organic cation transporter (OCT) 2 and multidrug and toxin extrusion proteins (MATE) 1 and 2‐K are of major importance. However, fully validated, sensitive, and specific biomarkers for these transporters are still lacking. In previous *in vivo* and *in vitro* studies, 5‐aminovaleric acid betaine (5AVAB), serotonin (5HT), and 1‐methylhistamine (1MH) emerged as promising biomarker candidates for OCT2/MATE‐mediated DDI. Therefore, we further investigated their suitability for clinical risk assessment by evaluating the effects of the OCT2/MATE inhibitor trimethoprim in healthy volunteers and by determining IC_50_ values for the inhibition of OCT2‐ and/or MATE1‐mediated uptake of the potential biomarkers by trimethoprim, cimetidine, pyrimethamine, and dolutegravir *in vitro*. Trimethoprim reduced the amount excreted into urine of 5AVAB, 5HT, and 1MH *in vivo* by 95.1 ± 5.67%, 78.9 ± 6.56%, and 51.9 ± 14.3%, respectively (all *P* < 0.001) as well as the renal clearance of 5AVAB and 5HT by 93.6 ± 5.85% and 73.8 ± 12.8%, respectively (both *P* < 0.001). When comparing the IC_50_ values with unbound inhibitor plasma concentrations, trimethoprim, cimetidine, and pyrimethamine preferentially inhibit the MATE1‐mediated transport, whereas dolutegravir inhibits the OCT2‐mediated transport of the biomarker candidates. In conclusion, this study further supports the suitability of 5‐aminovaleric acid betaine (5AVAB), serotonin (5HT), and 1‐methylhistamine (1MH) as promising biomarkers for OCT2/MATE‐mediated DDI.

**Study Highlights**

**WHAT IS THE CURRENT KNOWLEDGE ON THE TOPIC?**

The potential of a new drug to cause transporter‐mediated drug‐drug interactions (DDI) has to be evaluated during drug development. DDI risk assessment can be improved by the utilization of endogenous biomarkers. However, there is a lack of fully validated biomarkers for this purpose.

**WHAT QUESTION DID THIS STUDY ADDRESS?**

This study investigates the effects of trimethoprim, an inhibitor of the organic cation transporter (OCT) 2 and of the multidrug and toxin extrusion proteins (MATE) 1 and 2‐K, on the disposition of 5‐aminovaleric acid betaine (5AVAB), serotonin (5HT), and 1‐methylhistamine (1MH) in healthy volunteers. Furthermore, IC_50_ values for the inhibition of OCT2 and/or MATE1‐mediated biomarker transport by four clinically used OCT2/MATE inhibitors were determined.

**WHAT DOES THIS STUDY ADD TO OUR KNOWLEDGE?**

Trimethoprim significantly reduced the renal clearance and/or the amount excreted into urine of 5AVAB, 5HT, and 1MH. Similar findings were previously found with cimetidine, another inhibitor of OCT2/MATE. Therefore, the observed *in vivo* effects are most likely due to inhibition of the renal transporters OCT2 and/or MATE, rather than other effects of the inhibitors. These findings add novel insights into the use of 5AVAB, 5HT, and 1MH as biomarkers for OCT2/MATE‐mediated renal DDI *in vivo*.

**HOW MIGHT THIS CHANGE CLINICAL PHARMACOLOGY OR TRANSLATIONAL SCIENCE?**

The use of sensitive and specific biomarkers for transporter‐mediated DDI may decrease the number of necessary clinical trials, improve the clinical readouts, and improve the safety of drug use in patients.

Transport proteins determine cellular uptake and efflux of endogenous metabolites and exogenous compounds and are crucial determinants of drug disposition.^1^, ^2^, ^3^ Administration of drugs that interact with the same transporter may cause transporter‐mediated drug‐drug interactions (DDI), which can result in clinically important alterations of pharmacokinetic parameters and affect both desired and adverse drug effects.^1^, ^2^, ^3^, ^4^ Therefore, the recently published ICH M12 guideline on drug interaction studies, which was adopted by the EMA and the FDA, requests the assessment of potential transporter‐mediated DDI for transporters of emerging clinical relevance during early drug development.^5^ The guideline requests *in vitro* investigations to evaluate substrate or inhibitor properties of a new molecular entity, which may trigger clinical DDI studies.^5^ However, these studies are time and cost intensive, put healthy volunteers at risk and often do not reveal clinically relevant interactions.^6^, ^7^ Endogenous biomarkers received increasing attention as emerging tools for improving transporter‐mediated DDI risk assessment. Therefore, integrating endogenous biomarkers for transporter‐mediated DDI could help to reduce the number of such studies and improve clinical readouts.^5^, ^6^, ^7^, ^8^, ^9^, ^10^ However, only a limited number of biomarkers have been fully validated for clinical transporter‐mediated DDI risk assessment so far.^3^

With regard to the renal excretion of cationic drugs, the coordinated activity of the organic cation transporter (OCT) 2 (gene symbol *SLC22A2*) and the multidrug and toxin extrusion proteins (MATE) 1 and 2‐K (gene symbols *SLC47A1* and *SLC47A2*, respectively) is of major significance.^11^, ^12^ OCT2 is localized in the basolateral membrane of proximal tubule cells and mediates the uptake into the cell via facilitated diffusion, whereas MATE1 and MATE2‐K are proton‐exchanging antiporters localized on the apical side of the tubule cells mediating the efflux into urine.^13^, ^14^, ^15^ Substrates of OCT2/MATE are, for example, metformin and lamivudine, as well as the endogenous compounds creatinine and N^1^‐methylnicotinamide (NMN).^3^, ^16^, ^17^, ^18^

In previous work, we identified new biomarker candidates for renal transporter‐mediated DDI via OCT2/MATE based on a metabolomic analysis of human plasma and urine samples after treatment with four classical transporter inhibitors.^19^ Five biomarker candidates were further characterized as substrates of OCT2 and MATE1 *in vitro* and the transport mediated by other clinically relevant transport proteins (organic anion transporters 1 and 3, organic anion transporting polypeptides 1B1 and 1B3, P‐glycoprotein) was evaluated.^20^ Based on our previous *in vivo* and *in vitro* studies, 5‐aminovaleric acid betaine (5AVAB), serotonin (5HT), and 1‐methylhistamine (1MH) resulted as promising biomarker candidates for OCT2/MATE‐mediated DDI. 5AVAB, 5HT, and 1MH showed pronounced changes of the *in vivo* disposition due to cimetidine in healthy volunteers as well as high transport rates for OCT2‐ and/or MATE1‐mediated transport, without or only with minor transport by other renal transport proteins.^19^, ^20^

The goal of the present study was therefore to further evaluate the suitability of 5AVAB, 5HT and 1MH as potential biomarkers for OCT2/MATE‐mediated renal DDI *in vivo* and *in vitro*. For this purpose, we investigated the effects of trimethoprim on the *in vivo* disposition of 5AVAB, 5HT and 1MH in healthy volunteers by quantifying these potential biomarkers in plasma and urine samples from a previously published clinical DDI study.^17^ Trimethoprim is an inhibitor of the renal transporters OCT2, MATE1, and MATE2‐K (*K*_*i*_ values 27.2 ± 2.8 μM, 6.3 ± 2.0 μM and 28.9 ± 4.9 μM, respectively), which has been used in several clinical DDI studies assessing OCT2/MATE‐mediated DDI.^17^, ^21^, ^22^ Moreover, we determined IC_50_ values for the *in vitro* inhibition of OCT2‐ and/or MATE1‐mediated 5AVAB, 5HT and 1MH uptake by trimethoprim and other OCT2/MATE inhibitors (cimetidine, pyrimethamine, dolutegravir).^21^ These results provide novel insights into the suitability of 5AVAB, 5HT and 1MH as potential biomarkers for OCT2/MATE‐mediated DDI risk assessment.

## MATERIALS AND METHODS

A list of the materials used, a description of the LC–MS quantification of the potential biomarkers in human samples and in cell lysates, as well as cell culture conditions can be found in the **Supporting Information**. Furthermore, a summary of pharmacokinetic properties of trimethoprim, as well as its DDI characteristics, can be found in **Table** **S4**.

### Clinical study

Plasma and urine samples were collected during a single‐center, randomized, open‐label cross‐over study investigating the DDI of trimethoprim and metformin in 12 healthy male (8) and female (4) volunteers, previously published by Müller et al.^17^ Briefly, the study had two phases (A and B) separated by a wash out period of ≥ 7 days. Study time 0 hours was defined as the intake of the second dose of metformin. In phase A, subjects received metformin (850 mg) at the time points −12 hours (not fasted) and 0 hours (fasted). In phase B, metformin was administered at the same time points as in phase A and additionally, the subjects received trimethoprim (200 mg) twice daily for 5 days (at −96, −84, −72, −60, −48, −36, −24, −12, 0, and 12 hours). Food intake was controlled with a target carbohydrate intake of 200–250 g/day. Plasma samples were collected at 0 hours pre‐dose and at 0.5, 1, 1.5, 2, 2.5, 3, 3.5, 4, 5, 6, 8, 10, 12, 23.5, and 24 hours. Urine samples were collected in three intervals: 0–6, 6–12, and 12–24 hours. All samples were stored at −80°C prior to LC–MS analysis.

All performed analyses are in line with the written informed consent provided by the participants. The study was registered in the German Clinical Trials Register (ID: DRKS00004349) and was approved by the Ethics Committee of the Friedrich‐Alexander‐Universität Erlangen‐Nürnberg (Erlangen, Germany) and the Federal Institute for Drugs and Medical Devices (Bundesinstitut für Arzneimittel und Medizinprodukte; Bonn, Germany; EudraCT ID: 2012‐000500‐15).

### Uptake and inhibition assays in HEK cells

Uptake and inhibition assays were performed in human embryonic kidney 293 (HEK) cells stably overexpressing human OCT2 (HEK‐OCT2) or MATE1 (HEK‐MATE1) and in HEK cells stably transfected with an empty vector (HEK‐VC) as previously described with minor modifications.^23^, ^24^ Briefly, cells were seeded into 12‐well plates coated with poly‐D‐lysine (0.1 mg/mL) with an initial density of 7 × 10^5^ cells per well. After 24 hours, protein expression was enhanced using medium supplemented with sodium butyrate (10 mM). Transport assays were performed 48 hours after seeding. Cells were washed once with 37°C prewarmed uptake buffer (142 mM NaCl, 5 mM KCl, 1 mM K_2_HPO_4_, 1.2 mM MgSO_4_, 1.5 mM CaCl_2_, 5 mM glucose, and 12.5 mM HEPES in water). For experiments with HEK‐OCT2 a pH of 7.3 was used, whereas pH 8.0 was used for HEK‐MATE1 cells.^25^ Afterwards, cells were incubated for 5 minutes with 37°C prewarmed uptake buffer containing the respective substrate (mixture of [^2^H_9_]‐5AVAB and unlabeled 5AVAB, mixture of [^3^H]‐5HT and unlabeled 5HT or unlabeled 1MH) with or without inhibitor (trimethoprim, cimetidine, pyrimethamine, or dolutegravir). In HEK‐OCT2 cells, 5AVAB, 5HT, or 1MH were used as substrates at a concentration of 100 μM, whereas in HEK‐MATE1 cells, 5HT and 1MH were investigated at a concentration of 20 and 100 μM, respectively. Experiments assessing the inhibition of MATE1‐mediated 5AVAB transport were not performed, because previous data did not show an uptake of 5AVAB in HEK‐MATE1 cells.^20^

In experiments with 5AVAB and 1MH as substrates, cells were put on ice after incubation, the incubation buffer was removed, and the cells were washed three times with ice‐cold 0.9% saline. Thereafter, cells were lysed with 700 μL of an ice‐cold mixture of methanol (MeOH) and water (80:20) containing the recovery standards [^2^H_5_]‐tryptophan (1 μg/mL) and [^2^H_3_]‐1MH (25 ng/mL). Subsequently, the concentrations of [^2^H_9_]‐5AVAB or 1MH in the cell lysates were determined via LC–MS using calibration curves.

In experiments with 5HT, cells were put on ice after incubation, the incubation buffer was removed, and the cells were washed three times with ice‐cold uptake buffer (pH 7.3 or 8.0). Afterwards, the cells were lysed with 800 μL of 0.2% sodium dodecyl sulfate in water and the radioactivity in the samples was measured using liquid scintillation counting (Tri‐Carb™ 2800; Perkin Elmer Life and Analytical Sciences Inc., Rodgau‐Jügesheim, Germany or Tri‐Carb™ 4810TR; Revvity GmbH, Hamburg, Germany). The protein concentration in the samples was determined by bicinchoninic acid assay (Pierce™ BCA protein assay kit; Thermo Fischer Life Technologies GmbH, Darmstadt, Germany). The same procedures were applied for positive control experiments, for which a mixture of [^3^H]‐MPP^+^ and unlabeled MPP^+^ (10 μM), a well‐characterized OCT2/MATE substrate, was used as described previously.^20^, ^23^ The incubation time was 5 minutes for all control experiments. All *in vitro* experiments were performed with six biological replicates.

### Data analysis

#### *In vivo* data

Quantification of the plasma and urine concentrations of 5AVAB, 5HT and 1MH was performed using Sciex OS 4.0 (AB Sciex Ptc. Ltd.; Darmstadt, Germany). The area under the plasma concentration‐time curve over 24 hours (AUC_0–24h_) of 5AVAB and 5HT was calculated using the linear trapezoidal method in GraphPad Prism 10.6.1 (GraphPad Software; San Diego, USA), and the AUC_0–24h_ of metformin and NMN was calculated using Phoenix WinNonlin 6.3 (Pharsight; Mountain View, USA), as reported previously.^17^ The minimal (*C*_min_) and maximal (*C*_max_) plasma concentration was determined from individual plasma concentration‐time curves. The amount excreted into urine over 24 h (Ae_0–24h_) was calculated by multiplying the concentration found in urine by the respective urine volume. The renal clearance (Cl_R_) was calculated by dividing the Ae_0–24h_ by the plasma AUC_0–24h_. Statistical significance was analyzed by a two‐tailed paired Students' *t*‐test or a Wilcoxon matched‐pairs signed rank test as appropriate. A *P*‐value below 0.05 was considered statistically significant. The *P*‐values are reported as exact values; however, all *P*‐values smaller than 0.001 are presented as “*P* < 0.001”. Correlations were computed using the nonparametric Spearman correlation coefficient (*r*_S_). Statistical analyses were performed in GraphPad Prism 10.6.1. *In vivo* data are presented as mean ± standard deviation of the mean (SD).

#### *In vitro* data

Substrate concentrations found in the individual samples were normalized to the mean protein concentrations from the respective control experiments in experiments with 5AVAB and 1MH or to the protein concentration determined for the individual sample in experiments with 5HT. Net uptake was calculated by subtracting the mean uptake in HEK‐VC cells from the uptake found in OCT2‐ or MATE1‐overexpressing cells. For the determination of the half‐maximal inhibitory concentrations (IC_50_), the net uptake was presented in % of the net uptake without inhibitor, which was set to 100%. The IC_50_ values were calculated using a nonlinear variable slope (Hill slope) model in GraphPad Prism 10.6.1. *In vitro* data are presented as mean ± standard error of the mean (SEM).

## RESULTS

### In vivo

#### Effects of trimethoprim on the disposition of 5AVAB, 5HT, and 1MH

**Figure 1:**
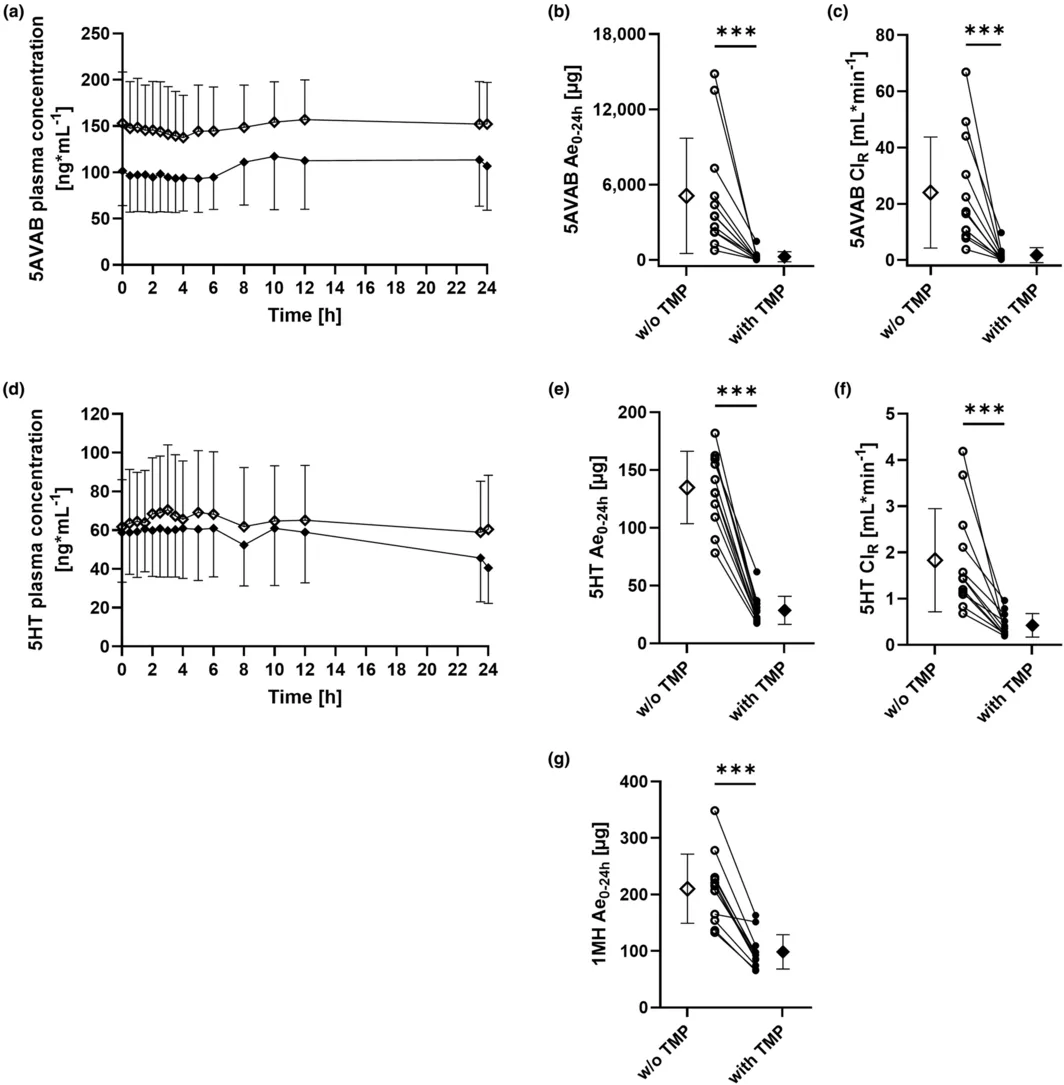
Effect of trimethoprim on the disposition of (**a–c**) 5‐aminovaleric acid betaine (5AVAB), (**d–f**) serotonin (5HT) and (**g**) 1‐methylhistamine (1MH) in healthy volunteers. (**a, d**) Plasma concentration‐time curve, (**b, e, g**) amount excreted into urine (Ae_0–24h_) and (**c, f**) renal clearance (Cl_R_) of the respective potential biomarker in the study phase without trimethoprim (w/o TMP; open symbols) and in the phase with trimethoprim (with TMP; filled symbols). Circles represent individual values, whereas diamonds represent group data, which are shown as mean ± standard deviation of the mean (SD). For better readability, SD is shown in one direction only in (**a, d**). ***P < 0.001 (phase w/o TMP vs. with TMP).

A summary of the pharmacokinetic parameters of 5AVAB, 5HT, and 1MH with and without trimethoprim is given in **Table** **1**. Plasma concentration‐time curves of 5AVAB and 5HT with and without trimethoprim are shown in **Figure** **1** **a,d**, respectively. Trimethoprim decreased the AUC_0–24h_ and *C*_max_ of 5AVAB by 29.9 ± 16.9% (*P* < 0.001) and 26.6 ± 24.4% (*P* = 0.006), respectively, whereas the AUC_0–24h_ and *C*_max_ of 5HT were not significantly altered by trimethoprim. The concentrations of 1MH in all plasma samples were below the limit of quantification of the LC–MS method. Trimethoprim reduced the Ae_0–24h_ of 5AVAB, 5HT, and 1MH by 95.1 ± 5.67%, 78.9 ± 6.56%, and 51.9 ± 14.3% (all *P* < 0.001), respectively (**Figure** **1** **b,e,g**). Furthermore, trimethoprim reduced the Cl_R_ of 5AVAB and 5HT by 93.6 ± 5.85% and 73.8 ± 12.8% (both *P* < 0.001), respectively (**Figure** **1** **c,f**).

**Table 1:** Summary of the pharmacokinetic parameters of 5‐aminovaleric acid betaine, serotonin, and 1‐methylhistamine in 12 healthy volunteers without or with trimethoprim

Putative biomarker	Pharmacokinetic parameter	Phase A (without trimethoprim)	Phase B (with trimethoprim)	*P*‐value
5‐Aminovaleric acid betaine	AUC_0–24h_ [ng×h×mL^−1^]	3,625 ± 1,079	2,590 ± 1,089	< 0.001
	*C*_max_ [ng×mL^−1^]	170 ± 48.4	128 ± 61.1	0.006
	*C*_min_ [ng×mL^−1^]	134 ± 45.3	89.1 ± 34.5	< 0.001
	Ae_0–24h_ [μg]	5,104 ± 4,597	249 ± 407	< 0.001
	Cl_R_ [mL×min^−1^]	24.0 ± 19.7	1.74 ± 2.68	< 0.001
Serotonin	AUC_0–24h_ [ng×h×mL^−1^]	1,532 ± 663	1,331 ± 547	0.219
	*C*_max_ [ng×mL^−1^]	78.3 ± 34.8	73.3 ± 28.1	0.466
	*C*_min_ [ng×mL^−1^]	51.9 ± 25.8	35.1 ± 15.0	0.021
	Ae_0–24h_ [μg]	135 ± 31.4	28.6 ± 12.2	< 0.001
	Cl_R_ [mL×min^−1^]	1.83 ± 1.12	0.424 ± 0.254	< 0.001
1‐Methylhistamine	Ae_0–24h_ [μg]	210 ± 61.2	98.5 ± 30.5	< 0.001

Data are shown as mean ± standard deviation of the mean. Ae_0–24h_, amount excreted into urine; AUC_0–24h_, area under the plasma concentration‐time curve; Cl_R_, renal clearance; *C*_max_, maximal plasma concentration; *C*_min_, minimal plasma concentration.

#### Correlation between Cl_R_ ratios of the potential biomarkers with metformin or NMN

**Figure 2:**
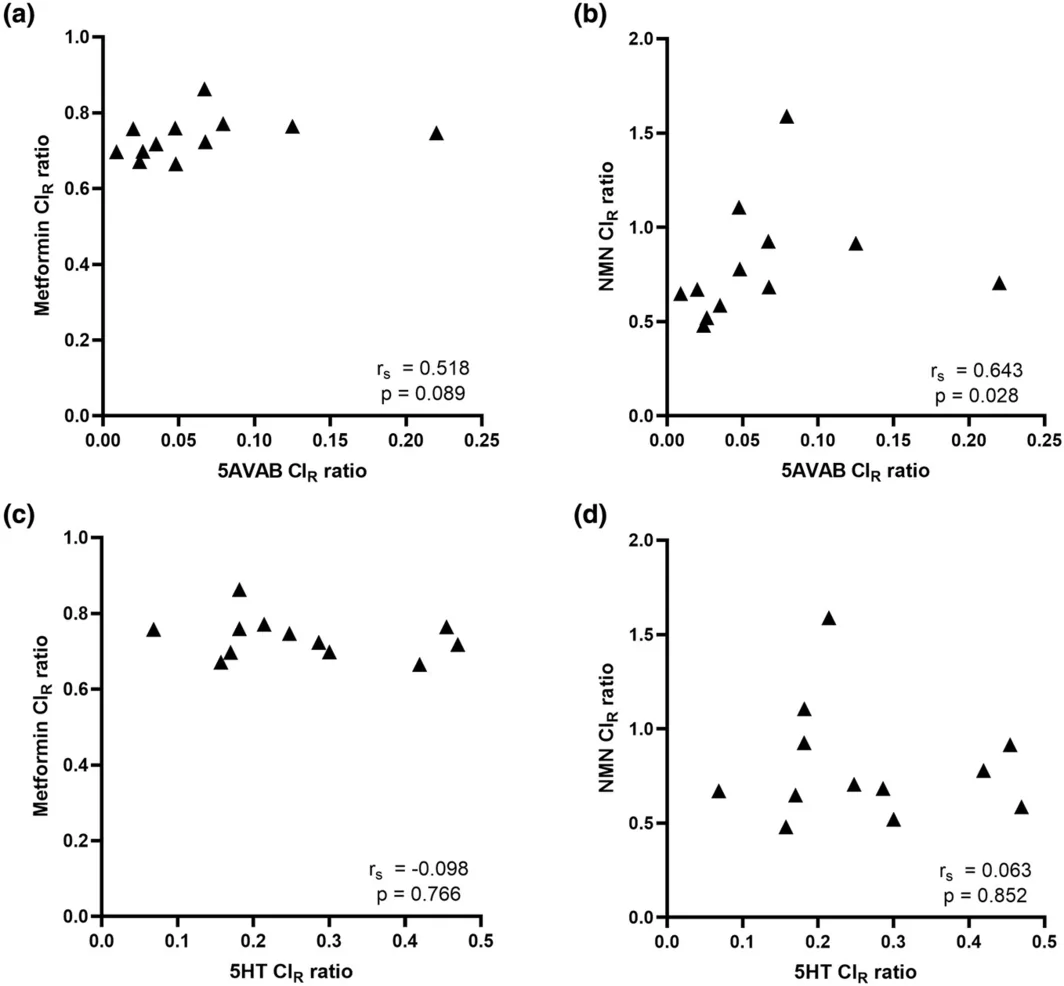
Correlation between the renal clearance (Cl_R_) ratios of (**a, c**) metformin or (**b, d**) N^1^‐methylnicotinamide (NMN) with Cl_R_ ratios of (**a, b**) 5‐aminovaleric acid betaine (5AVAB) or (**c, d**) serotonin (5HT). Cl_R_ ratios were calculated by dividing the individual Cl_R_ found in the phase with trimethoprim by the Cl_R_ from the phase without trimethoprim. Correlations were computed using the Spearman correlation coefficient (r_S_). Data for the Cl_R_ of metformin and NMN have been previously reported.^17^

Correlations between the changes of the Cl_R_ due to trimethoprim (ratio of phase with trimethoprim/without trimethoprim) of 5AVAB or 5HT with metformin or NMN are shown in **Figure** **2**. Data for the Cl_R_ of metformin and NMN were previously reported.^17^ The change of the Cl_R_ of 5AVAB showed a positive correlation with the change of the Cl_R_ of metformin; however, it was not statistically significant (*r*_S_ = 0.518, *P* = 0.089). Furthermore, the change of the Cl_R_ of 5AVAB due to trimethoprim showed a statistically significant, positive correlation with the Cl_R_ ratios of NMN (*r*_S_ = 0.643, *P* = 0.028). Correlations between the Cl_R_ ratios of 5HT and metformin (*r*_S_ = −0.098, *P* = 0.766) or NMN (*r*_S_ = 0.063, *P* = 0.852) appeared negligible. Correlations between the trimethoprim‐induced changes in the Cl_R_ of 1MH with metformin or NMN were not assessed, because the plasma concentrations of 1MH were below the limit of quantification of our LC–MS method, and therefore, the Cl_R_ of 1MH could not be calculated.

### *In vitro* inhibition experiments

For each *in vitro* inhibition experiment in OCT2‐ or MATE1‐overexpressing HEK cells, positive control experiments with the prototypical substrate MPP^+^ (10 μM) were performed. The respective results are shown in **Figure** **S1**.

#### Inhibition of the OCT2‐ and/or MATE1‐mediated uptake of 5AVAB, 5HT, and 1MH by trimethoprim

**Figure 3:**
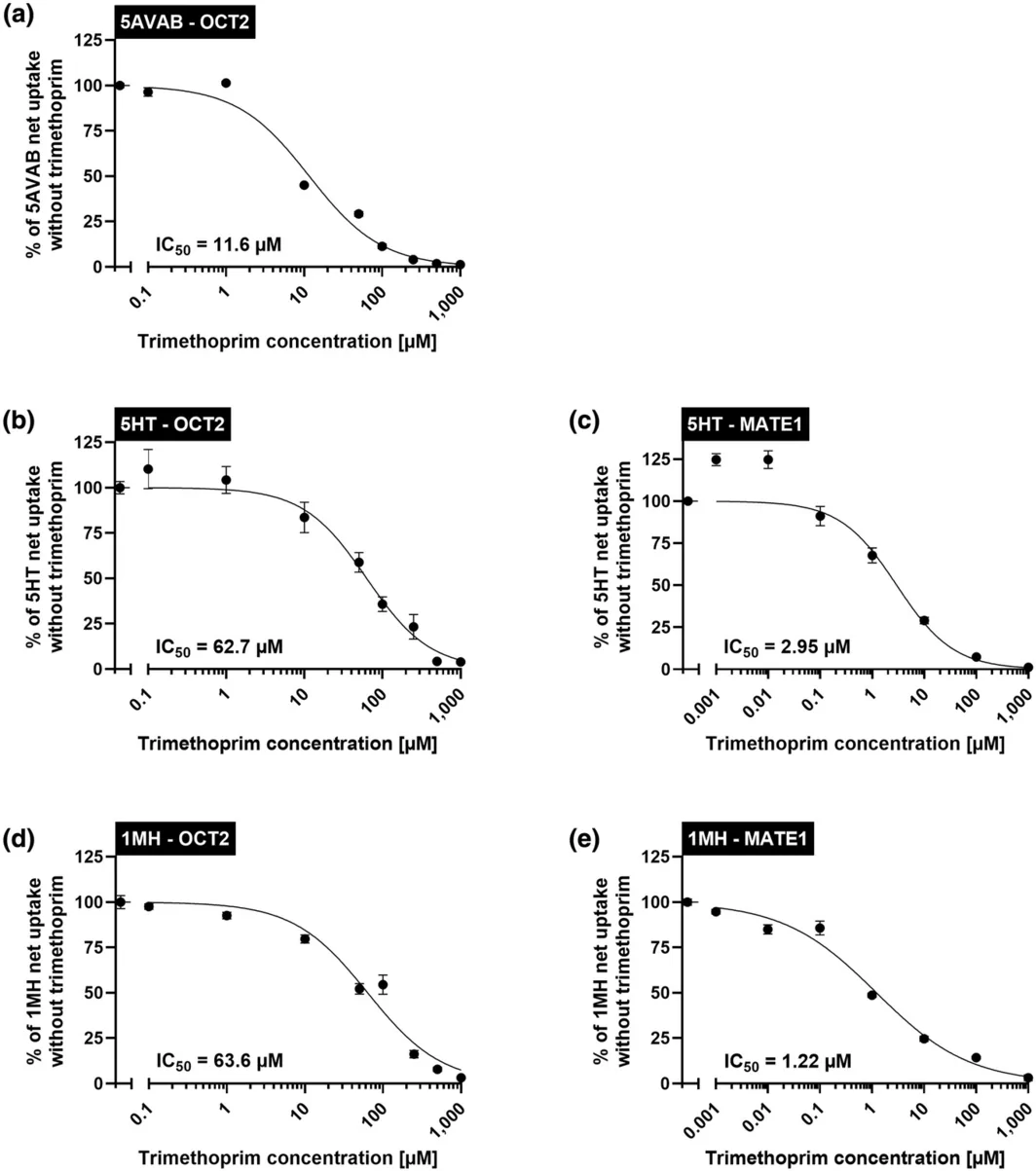
Determination of the half‐maximal inhibitory concentrations (IC_50_) for the inhibition of (**a**) OCT2‐mediated 5‐aminovaleric acid betaine (5AVAB; 100 μM) uptake, (**b**) OCT2‐mediated serotonin (5HT; 100 μM) uptake, (**c**) MATE1‐mediated 5HT (20 μM) uptake, (**d**) OCT2‐mediated 1‐methylhistamine (1MH; 100 μM) uptake, and (**e**) MATE1‐mediated 1MH (100 μM) uptake by trimethoprim. 5AVAB was not taken up in HEK‐MATE1 cells in a previous study.^20^ Data are shown in % of the net uptake without trimethoprim (set to 100%) as mean ± standard error of the mean (SEM) of six biological replicates. Net uptake was calculated by subtracting the uptake into vector control cells from the uptake into transporter‐overexpressing cells. In case of small SEM, error bars are partially hidden behind the data point symbols.

Results for the inhibition of the OCT2‐ and/or MATE1‐mediated uptake of the potential biomarkers by trimethoprim are shown in **Figure** **3**. The determined IC_50_ values for the inhibition of OCT2‐mediated 5AVAB, 5HT, and 1MH uptake by trimethoprim were 11.6, 62.7, and 63.6 μM, respectively. The MATE1‐mediated uptake of 5HT and 1MH was inhibited by trimethoprim with IC_50_ values of 2.95 and 1.22 μM, respectively. In a previous study, 5AVAB was shown not to be taken up into HEK‐MATE1 cells; therefore, a determination of the respective IC_50_ values was not possible.^20^

#### Inhibition of the OCT2‐mediated uptake of 5AVAB, 5HT, and 1MH by cimetidine, pyrimethamine, and dolutegravir

**Figure 4:**
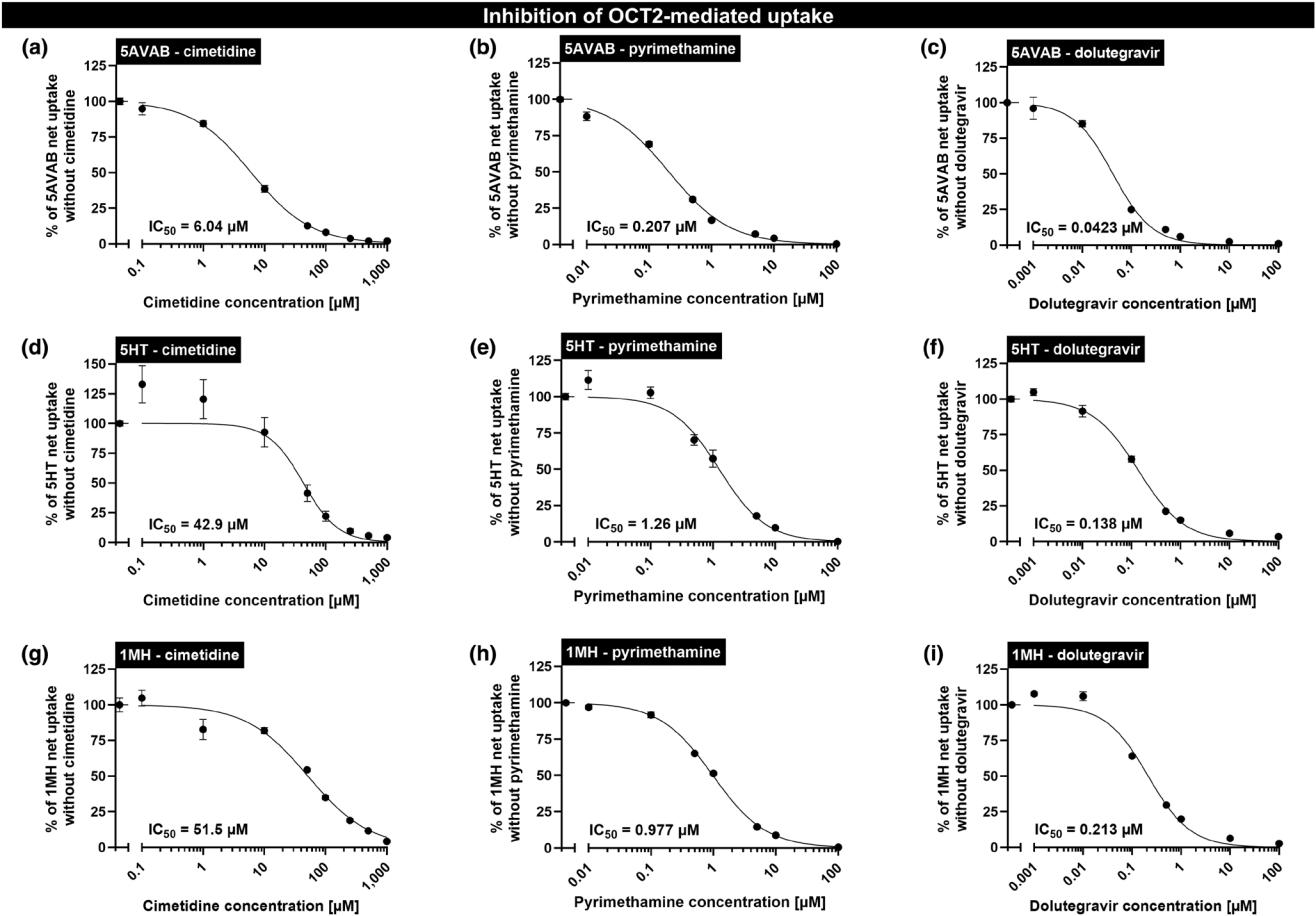
Determination of half‐maximal inhibitory concentrations (IC_50_) for the inhibition of OCT2‐mediated (**a–c**) 5‐aminovaleric acid betaine (5AVAB; 100 μM), (**d–f**) serotonin (5HT; 100 μM) or (**g–i**) 1‐methylhistamine (1MH; 100 μM) uptake by (**a, d, g**) cimetidine, (**b, e, h**) pyrimethamine and (**c, f, i**) dolutegravir. Data are shown in % of the net uptake without the respective inhibitor (set to 100%) as mean ± standard error of the mean (SEM) of six biological replicates. Net uptake was calculated by subtracting the uptake into vector control cells from the uptake into OCT2‐overexpressing cells. In case of small SEM, error bars are partially hidden behind the data point symbols.

Results for the inhibition of the OCT2‐mediated uptake of the potential biomarkers by cimetidine, pyrimethamine, and dolutegravir are shown in **Figure** **4**. The determined IC_50_ values for the inhibition of OCT2‐mediated uptake of 5AVAB, 5HT, and 1MH by cimetidine were 6.04, 42.9, and 51.5 μM, respectively; by pyrimethamine 0.207, 1.26, and 0.977 μM, respectively; and by dolutegravir 0.0423, 0.138, and 0.213 μM, respectively.

#### Inhibition of the MATE1‐mediated uptake of 5HT and 1MH by cimetidine, pyrimethamine and dolutegravir

**Figure 5:**
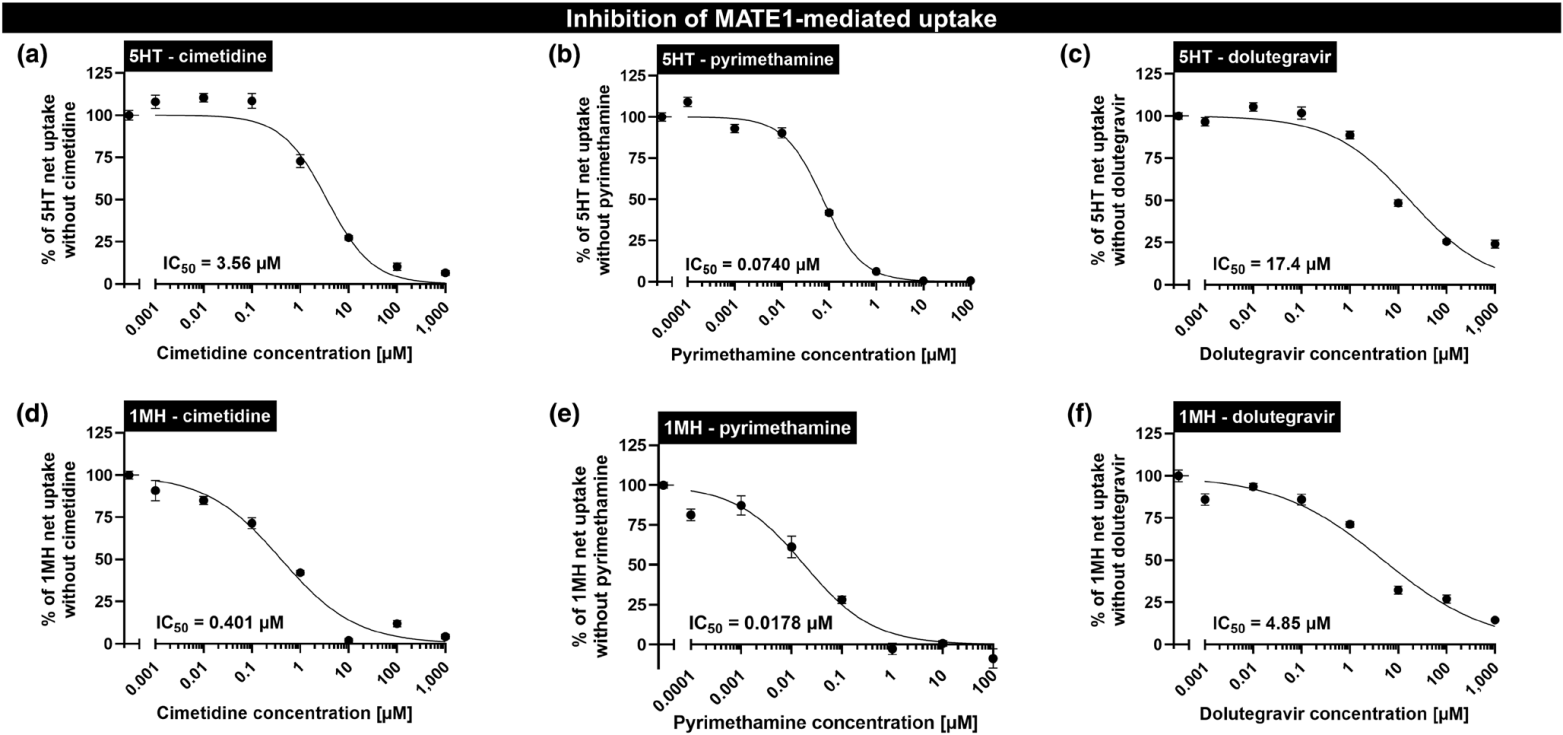
Determination of half‐maximal inhibitory concentrations (IC_50_) for the inhibition of MATE1‐mediated (**a–c**) serotonin (5HT; 20 μM) or (**d–f**) 1‐methylhistamine (1MH; 100 μM) uptake by (**a, d**) cimetidine, (**b, e**) pyrimethamine and (**c, f**) dolutegravir. 5AVAB was not taken up in HEK‐MATE1 cells in a previous study.^20^ Data are shown in % of the net uptake without the respective inhibitor (set to 100%) as mean ± standard error of the mean (SEM) of six biological replicates. Net uptake was calculated by subtracting the uptake into vector control cells from the uptake into MATE1‐overexpressing cells. In case of small SEM, error bars are partially hidden behind the data point symbols.

Results for the inhibition of the MATE1‐mediated uptake of 5HT and 1MH by cimetidine, pyrimethamine and dolutegravir are shown in **Figure** **5**. The determined IC_50_ values for the inhibition of MATE1‐mediated uptake of 5HT and 1MH by cimetidine were 3.56 and 0.401 μM, respectively; by pyrimethamine 0.0740 and 0.0178 μM, respectively; and by dolutegravir 17.4 and 4.85 μM, respectively. Determination of the respective IC_50_ values for the MATE1‐mediated 5AVAB uptake was not possible, because 5AVAB was not taken up into HEK‐MATE1 cells as described previously.^20^

## DISCUSSION

The present work focused on the effects of trimethoprim on the disposition of 5AVAB, 5HT, and 1MH in healthy volunteers, as well as on the effect of four classical OCT2/MATE inhibitors on the OCT2‐ and/or MATE1‐mediated transport of these potential biomarkers *in vitro* and thus extends the knowledge about the suitability of 5AVAB, 5HT, and 1MH as biomarkers for OCT2/MATE‐mediated renal DDI. The major findings of this study were as follows: (1) trimethoprim significantly reduces the Cl_R_ of 5AVAB and 5HT, as well as the Ae_0–24h_ of 5AVAB, 5HT, and 1MH in healthy volunteers; (2) the trimethoprim‐induced magnitude of change in the Cl_R_ of 5AVAB and NMN shows a moderate positive correlation; (3) based on the comparison of IC_50_ values with unbound inhibitor plasma concentrations, trimethoprim, cimetidine, pyrimethamine, and dolutegravir could be expected to show effects on the disposition of 5AVAB, 5HT, and 1MH *in vivo* due to inhibition of OCT2 and/or MATE1.

In this study, trimethoprim significantly reduced the Ae_0–24h_ of 5AVAB, 5HT, and 1MH. This is in line with our previous findings from an untargeted metabolomic study, where the urinary excretion of all three potential biomarkers showed a sensitive decrease after treatment with cimetidine.^19^ The consistency of the effects obtained with trimethoprim and cimetidine further strengthens our hypothesis that the observed *in vivo* effects are due to inhibition of OCT2/MATE rather than other effects of the inhibitors.

Trimethoprim significantly reduced the AUC_0–24h_, *C*_max_, and *C*_min_ of 5AVAB. Similarly, a decrease of the *C*_max_ was previously shown for NMN, another potential biomarker for OCT2/MATE‐mediated renal DDI.^17^, ^26^ A possible explanation is the inhibition of transporter‐mediated efflux from the site of biosynthesis or storage into the systemic circulation by trimethoprim (or cimetidine), a mechanism that has been previously proposed for other endogenous compounds as well.^27^, ^28^ An inhibition of 5AVAB biosynthesis, possibly due to the antimicrobial properties of trimethoprim, cannot be excluded as a reason for the observed decrease in the AUC_0–24h_. However, an inhibition of biosynthesis alone would not result in a pronounced reduction in the Cl_R_ of 5AVAB as described below.

Moreover, the Cl_R_ of both 5AVAB and 5HT were significantly decreased due to trimethoprim. The Cl_R_ of 5AVAB was reduced by 93.6 ± 5.85% despite the concomitant decrease in the AUC_0–24h_, indicating a considerable decline in the urinary excretion of 5AVAB due to trimethoprim. For 5HT, trimethoprim reduced the Cl_R_ by 73.8 ± 12.8%. These reductions of the Cl_R_ are more pronounced than those reported for NMN and creatinine, other proposed biomarkers for OCT2/MATE‐mediated DDI, where trimethoprim caused a decline in the Cl_R_ of each of them by approximately 20%.^17^, ^29^ Compounds eliminated via tubular secretion often show a Cl_R_ exceeding the glomerular filtration rate of the kidney. In the present study; however, the absolute values determined for the Cl_R_ of 5AVAB and 5HT were considerably lower, suggesting that other processes are involved in the renal elimination of 5AVAB and 5HT as well, such as reabsorption or metabolism. Although the Cl_R_ of 5AVAB and 5HT was low, a decrease in the renal elimination observed with the two OCT2/MATE inhibitors trimethoprim and cimetidine in independent studies strongly suggests a significant contribution of tubular secretion via OCT2/MATE to the overall renal excretion.

A quantitative correlation of the magnitude of inhibitor‐induced change between the biomarker and a victim drug would increase the predictive value of a biomarker. Although statistically significant correlations between the biomarkers and metformin were not observed in our study, the data suggest a moderate positive correlation of the trimethoprim‐induced change in Cl_R_ between 5AVAB and metformin (*r*_S_ = 0.518, *P* = 0.089). Moreover, a statistically significant moderate positive correlation of the Cl_R_ ratio was found between 5AVAB and NMN (*r*_S_ = 0.643, *P* = 0.028). Given that Cl_R_ changes of NMN due to trimethoprim have previously been shown to significantly correlate with those of metformin (*r*_S_ = 0.727, *P* = 0.010), this finding further supports the relevance of 5AVAB as a potential biomarker for OCT2/MATE‐mediated DDI.^17^ While metformin is recommended by the ICH M12 guideline as a probe drug for the assessment of OCT2/MATE‐mediated DDI, its pharmacokinetics is complex and several processes are involved (see **Table** **S6** for an overview).^5^, ^30^ Those processes, as well as the substrate recognition by the transporters, may differ for metformin and the potential biomarkers and thus impact correlations. Larger studies are needed to assess correlations between the Cl_R_ ratios of metformin and/or NMN with the potential biomarkers in more detail.

A comparison of the determined IC_50_ values with the unbound plasma concentration of the inhibitor can be used to predict the possible inhibitor effect on the biomarker disposition *in vivo*. For trimethoprim, the previously estimated unbound plasma concentrations in our study were 4–9 μM.^17^ Taking these concentrations into account, the decrease of the Ae_0–24h_ of 5HT and 1MH is likely due to the preferential inhibition of MATE1 rather than OCT2. A more pronounced effect of trimethoprim on the OCT2‐mediated transport could be expected for 5AVAB (IC_50_ = 11.6 μM). The determined IC_50_ values for the inhibition of OCT2‐ and/or MATE1‐mediated uptake of 5AVAB, 5HT, and 1MH by trimethoprim are lower than the ones described previously for the inhibition of NMN uptake, suggesting a higher sensitivity of 5AVAB, 5HT, and 1MH to inhibition of OCT2 and/or MATE1 by trimethoprim.^17^

Previously described unbound plasma concentrations of cimetidine, pyrimethamine and dolutegravir in humans were 4–21, 0.3, and 0.07–0.2 μM, respectively.^31^, ^32^, ^33^ At these concentrations, cimetidine and pyrimethamine would preferentially inhibit the MATE1‐mediated transport of 5HT and 1MH, whereas the OCT2‐mediated 5AVAB, 5HT, and 1MH uptake would be preferentially inhibited by dolutegravir, which is in accordance with previous findings.^21^ Nevertheless, cimetidine and pyrimethamine also showed a pronounced effect on the OCT2‐mediated transport of 5AVAB (IC_50_ = 6.04 and 0.207 μM, respectively), as mentioned above for trimethoprim. Furthermore, this comparison suggests that pyrimethamine and dolutegravir, in addition to trimethoprim and cimetidine, could also induce measurable effects on the biomarker disposition *in vivo* due to the inhibition of OCT2 and/or MATE1.

Important traits of a biomarker for transporter‐mediated DDI are high sensitivity, specificity, and robustness.^6^ Regarding sensitivity and specificity, we have previously shown that 5AVAB, 5HT, and 1MH are sensitive and specific substrates of OCT2 and MATE1 *in vitro*.^20^ Furthermore, in an untargeted metabolomics study, we previously demonstrated that 5HT and 1MH show a sensitive and specific decrease in the urinary excretion due to cimetidine, whereas 5AVAB was classified as sensitive and potentially specific for OCT2/MATE inhibition.^19^ The current study shows that trimethoprim has similar effects to cimetidine, demonstrating a consistent sensitive biomarker response to OCT2/MATE inhibition.

In our study, the plasma concentration of 5AVAB does not seem to be impacted by a circadian rhythm. Furthermore, no changes of the 5AVAB plasma concentration were detected after food intake within our study. In another study, the impact of a healthy nordic diet on the plasma concentrations of betaines was evaluated and plasma concentrations of 5AVAB did not show significant changes compared to the control group.^34^ Although 5AVAB is a microbiota‐derived metabolite, it is also contained in milk and meat.^35^, ^36^ The precursor of 5AVAB, trimethyllysine, was found in whole grain products, therefore a long‐term impact of diet has to be further evaluated.^35^

For 5HT, we did not find a circadian rhythm of the plasma concentration in our study, either. However, 5HT in men shows a seasonal variability of plasma concentrations with a peak during summer, but to the best of our knowledge, a comprehensive characterization of its circadian profile in humans has not yet been established.^37^ In rats; however, 5HT concentrations in plasma increase during daytime with a peak in the evening and decrease during the night.^38^ In case of clinical DDI studies, the knowledge of the circadian rhythm would be essential to determine the optimal sampling timepoints for biomarker quantification. With regard to biosynthesis, 5HT is a metabolite of the amino acid tryptophan and about 95% of the peripheral 5HT is produced in enterochromaffin cells of the gastrointestinal tract and after release into the circulation, it is taken up into the platelets via the 5HT transporter (SERT), where almost the total whole blood 5HT is stored.^39^ This has to be taken into account during analysis, since the concentrations of 5HT may differ substantially between plasma and whole blood.^40^ 5HT can be further metabolized to 5‐hydroxyindoleacetic acid via the monoamine oxidase or to melatonin via enzymatic acetylation and methylation.^41^ This makes the kinetic parameters of 5HT susceptible to inhibition, induction or polymorphisms of the respective enzymes. Therefore, Cl_R_ appears as the most useful parameter with respect to potential transporter‐mediated renal DDI.

In our study, 1MH could be quantified in urine samples only; therefore, we were not able to evaluate a potential circadian rhythm in plasma. To the best of our knowledge, diurnal changes of 1MH in plasma have not been reported in the literature. 1MH is a metabolite of histamine, which is produced from the amino acid histidine.^36^ The urinary levels of 1MH were shown to be increased in patients after anaphylactic reactions and decreased in adult patients with histamine intolerance compared to healthy controls.^42^, ^43^ 1MH can be further metabolized to 1‐methylimidazoleacetic acid via a two‐step enzymatic oxidation.^41^ Taking this into account, the Cl_R_ would be the most suitable parameter with respect to OCT2/MATE‐mediated DDI. However, the quantification of 1MH in plasma appears analytically challenging and may necessitate the use of the Ae_0–24h_ for DDI detection. The Ae_0–24h_; however, is dependent on histamine levels and should therefore be used with caution for DDI risk assessment, ideally as a part of a broader panel of biomarkers. A summary of available information about biosynthesis, metabolism and transport pathways regarding 5AVAB, 5HT, and 1MH can be found in **Table** **S5** in the **Supporting Information**.

So far, there are no fully validated biomarkers available for OCT2/MATE‐mediated risk assessment; only NMN and creatinine have been partially validated, and for example, N^1^‐methyladenosine (m1A) is considered exploratory.^3^ The major advantage of NMN, creatinine, and m1A is the data availability from clinical studies in humans for different OCT2/MATE inhibitors, as well as significant quantitative correlations between the biomarkers and the victim drug metformin.^17^, ^18^, ^31^, ^44^, ^45^, ^46^ A summary of strengths and weaknesses of the use of metformin as a probe drug, as well as of 5AVAB, 5HT, 1MH, and the partially validated biomarkers NMN and creatinine can be found in **Table** **S6** in the **Supporting Information**. Although NMN and creatinine are better characterized, 5AVAB and 5HT show considerably higher changes in the Cl_R_ due to trimethoprim, as mentioned above. However, the relative contribution of renal filtration, secretion, and reabsorption to the overall clearance of 5AVAB and 5HT is unknown.

For the present study, some limitations need to be considered. Trimethoprim may have other effects impacting biosynthesis, transport or metabolism of 5AVAB, 5HT, and 1MH, which might affect the disposition of these biomarker candidates, for instance due to its antimicrobial properties or inhibitory effect on some cytochrome P450 enzymes.^47^ To further investigate the suitability of 5AVAB, 5HT, and 1MH as biomarkers for OCT2/MATE‐mediated renal DDI, studies with a larger sample size (i.e., a larger number of participants), as well as with other inhibitors of OCT2/MATE should be performed. Furthermore, the data on the disposition of 1MH *in vivo* are limited due to the sensitivity of the used LC–MS method. A more sensitive method for quantifying 1MH in plasma samples would be useful to assess the effect of OCT2/MATE inhibitors on the AUC_0–24h_ and Cl_R_ of 1MH.

Regarding *in vitro* experiments, the transport direction of MATE1 was reversed by adjusting the extracellular pH to 8.0 to enable the assessment of intracellular accumulation. Although it was recently shown that the transport direction of MATE1 does not substantially impact inhibition, this procedure may not be suitable for all kinds of substrates.^48^ Furthermore, the substrate concentrations of 5AVAB and 1MH were not always below the respective K_m_ values for the transporter, as recommended by the ICH M12 guideline, since the OCT2‐mediated transport of 5AVAB and 1MH was not saturable and therefore determination of exact K_m_ values was not possible (**Figure** **S2** and Gessner et al., 2025).^5^, ^20^ The selection of substrate concentrations of 5AVAB and 1MH used in inhibition experiments in HEK‐OCT2 cells was therefore based on robust and precise quantification via LC–MS in cell lysates. Furthermore, we did not investigate the MATE2‐K‐mediated transport of 5AVAB, 5HT, and 1MH *in vitro*, although trimethoprim is a known inhibitor of MATE2‐K as well.^21^, ^33^ However, the protein expression of MATE2‐K in the human kidney was reported to be very low compared to MATE1, suggesting MATE1 has a higher relevance in the excretion of organic cations.^49^ Nevertheless, a contribution of MATE2‐K to the renal excretion of 5AVAB, 5HT, and 1MH cannot be ruled out.

In conclusion, trimethoprim caused sensitive changes in the disposition of 5AVAB, 5HT, and 1MH in healthy volunteers and together with previous *in vivo* and *in vitro* data further strengthens the hypothesis that the observed changes are due to inhibition of renal OCT2/MATE.^19^, ^20^ Furthermore, the determined IC_50_ values suggest that other OCT2/MATE inhibitors could lead to *in vivo* changes in the disposition of 5AVAB, 5HT, and 1MH as well. Taken together, 5AVAB, 5HT, and 1MH are sensitive and specific biomarker candidates for the assessment of OCT2/MATE‐mediated renal DDI. However, additional studies are needed to comprehensively evaluate the predictivity and the impact of confounding factors on the disposition of these biomarker candidates.

## FUNDING

The QTRAP 6500+ was funded by the Deutsche Forschungsgemeinschaft (DFG, German Research Foundation) – 537496341.

## CONFLICT OF INTEREST

F.M. is an employee of Boehringer Ingelheim. M.F.F. has received consultancy fees from Boehringer Ingelheim and lecture fees from Janssen‐Cilag. He has received third‐party funds for research projects at his institution from Boehringer Ingelheim and Heidelberg Pharma Research GmbH. M.F.F. and colleagues received an earmarked financial contribution for the first award of the MSD Germany Health Award 2021. All other authors declared no competing interests for this work.

## AUTHOR CONTRIBUTIONS

J.P. and A.G. wrote the manuscript; F.M., J.K., M.F.F., and A.G. designed the research; J.P. and D.A. performed the research; J.P., D.A., and A.G. analyzed the data.

## Supporting information


Table S1.
