# Supplementary material for: Effects of Trimethoprim on Three Previously Proposed Putative Biomarkers for OCT2/MATE‐Mediated Renal Drug‐Drug Interactions in Healthy Volunteers
Source: Clin Pharmacol Ther. 2026 Jul 4:10.1002/cpt.70384. Online ahead of print. doi: 10.1002/cpt.70384 (PMC13337132; doi:10.1002/cpt.70384)
Supplement: Supplementary file 1 — Table S1. [file CPT-9999-0-s001.pdf]

SUPPORTING INFORMATION

Effects of trimethoprim on three previously proposed putative biomarkers for OCT2/MATE-mediated renal drug-drug interactions in healthy volunteers

Jana Picurová[1], Fabian Müller[1,2], Daniel Auge[1], Jörg König[1,3], Martin F. Fromm[1,3] and Arne Gessner[1,3]

[1]Institute of Experimental and Clinical Pharmacology and Toxicology, Friedrich-Alexander-Universität Erlangen-Nürnberg, Erlangen, Germany

[2]Boehringer Ingelheim Pharma GmbH & Co. KG, Biberach an der Riss, Germany

[3]FAU NeW – Research Center New Bioactive Compounds, Friedrich-Alexander-Universität Erlangen-Nürnberg, Erlangen, Germany

Address for correspondence: Dr. Arne Gessner, Institute of Experimental and Clinical Pharmacology and Toxicology, Friedrich-Alexander-Universität Erlangen-Nürnberg, Fahrstr. 17, 91054 Erlangen, Germany. Tel: +49 9131 85 22869; E-mail: arne.gessner@fau.de

SUPPLEMENTARY METHODS

Materials

Cellstar® 12-well cell culture plates were acquired from Greiner Bio-One GmbH (Frickenhausen, Germany). Sodium butyrate, poly-D-lysine hydrobromide, ammonium formate (LC-MS grade), serotonin HCl, 1-methylhistamine HCl, trimethoprim, cimetidine, pyrimethamine, [$^2H_4$]-serotonin HCl and [$^2H_5$]-tryptophan were purchased from Merck KGaA (Darmstadt, Germany). 5-aminovaleric acid betaine, [$^2H_9$]-5-aminovaleric acid betaine and [$^2H_3$]-1-methylhistamine were acquired from MedChemExpress (Monmouth Junction, USA). 1-methyl-4-phenylpyridinium iodide and dolutegravir were from Biomol (Hamburg, Germany). [^3H]-1-methyl-4-phenylpyridinium iodide (80 Ci/mmol) was obtained from American Radiolabeled Chemicals, Inc. (St. Louis, USA). [^3H]-serotonin (80 Ci/mmol) was from Hartmann Analytic (Braunschweig, Germany). Methanol, water, acetonitrile and formic acid (all LC-MS grade) as well as 0.3 mL and 1.5 mL autosampler vials were obtained from VWR chemicals (Darmstadt, Germany). All cell culture media and supplements were obtained from Thermo Fischer Life Technologies GmbH (Darmstadt, Germany). Unless stated otherwise, all other reagents and chemicals were purchased from Carl Roth GmbH + Co. KG (Karlsruhe, Germany).

Determination of 5AVAB, 5HT and 1MH concentrations in plasma and urine samples

5AVAB, 5HT and 1MH in plasma and urine samples were quantified using a validated LC-MS method on an HPLC system (Shimadzu Nexera® CL UHPLC; Shimadzu Deutschland GmbH, Duisburg, Germany) coupled to a QTRAP® 6500+ MS system (AB Sciex Germany GmbH, Darmstadt, Germany) with an electrospray ion source operating in positive ionization mode. A gradient elution with 10 mmol/L ammonium formate in a mixture of acetonitrile and water (95:5) with 0.1% formic acid (eluent A) and 10 mmol/L ammonium formate in water with 0.1% formic acid (eluent B) was performed with a flow rate of 0.35 mL/min and a run time of 20 min.

The gradient program is shown in Table S1 and Fig. S3. For chromatographic separation a 2.1 x 100 mm Acquity® PRM BEH amide column (1.7 µm) with a guard-column (both from Waters, Eschborn, Germany) was used. The column temperature was set to 40 °C. The retention times and MRM transitions of the analytes are listed in Table S2.

Sample preparation: 20 µL of EDTA-plasma or urine samples were mixed with 2 µL water and 100 µL internal standard solution consisting of [$^2H_9$]-5AVAB (2 ng/mL), [$^2H_4$]-5HT (20 ng/mL) and [$^2H_3$]-1MH (10 ng/mL) in methanol, vortexed and subsequently centrifuged at 16,000 rpm and 4 °C for 5 min (Eppendorf 5427R centrifuge; Eppendorf, Hamburg, Germany). 100 µL of the supernatant were pipetted into an insert vial, of which 2 µL were injected for LC-MS analysis. Calibration standards were prepared by adding 2 µL of the standard dilution series to 20 µL of 5-times dialyzed pooled EDTA-plasma or synthetic urine (Synthetic urine DIN EN 1616; Synthetic Urine e.K., Eberdingen-Nussdorf, Germany), mixed with 100 µL internal standard solution and prepared as described above. The calibration ranges for the analytes are listed in Table S2.

Cell culture

Cells were cultivated in minimal essential medium supplemented with 10% heat-inactivated fetal bovine serum, 100 U/mL penicillin, 100 µg/mL streptomycin and either 260 µg/mL hygromycin B (HEK-OCT2) or 800 µg/mL geneticin (HEK-MATE1). Cells were cultured at 37 °C with 5% CO_2 and were subcultured as needed using trypsin-EDTA (0.05% - 0.02%) solution.

Determination of [$^2H_9$]-5AVAB and 1MH concentrations in cell lysates

[$^2H_9$]-5AVAB and 1MH in cell lysates were quantified by an HPLC system (Agilent® 1100; Agilent Technologies, Waldbronn, Germany) coupled to a Triple-Quadrupole-MS (API 4000™; Applied Biosystems, Darmstadt, Germany) with an electrospray ion source operating in positive ionization mode. Chromatography was carried out isocratically at a flow rate of

0.3 mL/min and a run time of 5.5 min. Eluent consisted of 10 mmol/L ammonium formate in a mixture of acetonitrile and water (80:20), the pH was adjusted to 3.5 with formic acid. For chromatographic separation a 2.1 x 100 mm Acquity® UPLC BEH amide column (1.7 µm) with a 2.1 x 5 mm guard-column (both from Waters, Eschborn, Germany) was used. The column temperature was set to 40 °C. The retention times and MRM transitions of the analytes are listed in Table S3.

Sample preparation: The methanolic cell lysates were centrifuged at 16,000 rpm at 4 °C for 5 min (Eppendorf 5427R centrifuge; Eppendorf, Hamburg, Germany). 200 µL of the supernatant were pipetted into a vial and evaporated to dryness at 30 °C under a stream of nitrogen. The residue was resolved in 300 µL eluent and 10 µL were injected for LC-MS analysis. Calibration standards were created by diluting the standard solutions with blank cell lysate matrix (ratio 1:10) and prepared as described above. The calibration ranges for the analytes are listed in Table S3.

SUPPLEMENTARY TABLES AND FIGURES

Table S1: Gradient program of the LC-MS method used for the quantification of 5-aminovaleric acid betaine, serotonin and 1-methylhistamine in plasma and urine samples

Time (min)	Flow rate (mL/min)	Concentration A (%)	Concentration B (%)	Curve B
0	0.350	100	0	0
0.5	0.350	100	0	2
7.80	0.350	30	70	0
8.10	0.350	30	70	0
8.30	0.350	100	0	0
14.00	0.350	100	0	0

Eluent A: 10 mmol/L ammonium formate in a mixture of acetonitrile and water (95:5) with 0.1% formic acid; eluent B: 10 mmol/L ammonium formate in water with 0.1% formic acid

Table S2: Retention times and MRM transitions of the method used for quantification of 5-aminovaleric acid betaine, serotonin and 1-methylhistamine in plasma and urine samples

**Analytes**	**Calibration range**	**RT (min)**	**Q1**	**Q3**
5-aminovaleric acid betaine (quantifier)	1 – 1,000 ng/mL (plasma) 5 – 5,000 ng/mL (urine)	5.9	160.1	101.1
5-aminovaleric acid betaine (qualifier)				55.1
serotonin (quantifier)	1 – 1,000 ng/mL (plasma and urine)	4.8	177.1	160.1
serotonin (qualifier)				115.1
1-methylhistamine (quantifier)	10 – 1,000 ng/mL (plasma) 1 – 1,000 ng/mL (urine)	5.9	126.1	109.1
1-methylhistamine (qualifier)				68.0

**Internal standards**	**RT (min)**	**Q1**	**Q3**
[$^2H_9$]-5-aminovaleric acid betaine	5.9	169.2	101.0
[$^2H_4$]-serotonin	4.8	181.1	164.1
[$^2H_3$]-1-methylhistamine	5.9	129.1	100.2

RT: retention time, Q1: precursor ion, Q3: product ion

Table S3: Retention times and MRM transitions of the method used for quantification of [$^2H_9$]-5-aminovaleric acid betaine and 1-methylhistamine in cell lysates

**Analytes**	**Calibration range**	**RT (min)**	**Q1**	**Q3**
[$^2H_9$]-5-aminovaleric acid betaine (quantifier)	500 pM – 1 µM	1.4	169.0	101.1
[$^2H_9$]-5-aminovaleric acid betaine (qualifier)				55.2
1-methylhistamine (quantifier)	500 pM– 500 nM	3.6	126.1	109.2
1-methylhistamine (qualifier)				68.0

**Recovery standards**	**RT (min)**	**Q1**	**Q3**
[$^2H_5$]-tryptophan	1.7	210.1	192.2
[$^2H_3$]-1-methylhistamine	3.6	128.9	68.0

RT: retention time, Q1: precursor ion, Q3: product ion

Table S4: Overview of pharmacokinetic properties and drug-drug interaction characteristics of trimethoprim

**ADME characteristics**	- complete absorption from the gastrointestinal tract[1–3]<br>- peak serum concentrations achieved between 1 – 4 h after intake[1–3]<br>- steady state within 72 h of daily administration[2]<br>- 45% plasma protein binding[1–3]<br>- 20% metabolized in the liver[1,3]<br>- apparent volume of distribution: 69 – 138 L[2,3]<br>- elimination half-life (normal renal function): 10 – 14 h[2]	
**Dosing regimen in the study by Müller et al.[4]**	- 200 mg twice daily for 5 days<br>- timepoints: -96 h, -84 h, -72 h, -60 h, -48 h, -36 h, -24 h, -12 h, 0 h, and 12 h (study time 0 h = intake of the second dose of metformin)	
**PK parameters previously reported by Müller et al.[4]**	- $C_{max,ss}$: 14.8 ± 2.7 µM<br>- $AUC_{0\text{-}12h,ss}$: 129 ± 25.3 µM*h	
**DDI characteristics (ratios with/without trimethoprim)**<br><br>for exact dosing regimens of trimethoprim and the respective victim drugs see the respective referenced original publication	metformin[4]	RC_{max}: 1.23<br>RAUC: 1.30<br>RCl_R: 0.736
	metformin[5]	RC_{max}: 1.38<br>RAUC: 1.37<br>RCl_R: 0.68
	procainamide[6]	RAUC: 1.63<br>RCl_R: 0.53
	lamivudine[7]	RC_{max}: 1.09<br>RAUC: 1.43<br>RCl_R: 0.65
	zidovudine[8]	RC_{max}: 0.85<br>RAUC: 1.32<br>RCl_R: 0.42

Data for $C_{max,ss}$ and $AUC_{0\text{-}12h,ss}$ are shown as mean ± SD. ADME: absorption, distribution, metabolism, elimination, PK: pharmacokinetics, DDI: drug-drug interaction, $C_{max,ss}$: maximal plasma concentration in steady state, $AUC_{0\text{-}12h,\,ss}$: area under the plasma concentration – time curve between 0 – 12 h in steady state, AUC: area under the plasma concentration – time curve, Cl_R: renal clearance, RC_{max}: ratio of maximal plasma concentration (with/without trimethoprim), RAUC: ratio of the area under the curve (with/without trimethoprim), RCl_R: ratio of the renal clearance (with/without trimethoprim)

Table S5: Summary of synthesis, metabolism and known transport pathways of 5-aminovaleric acid betaine, serotonin and 1-methylhistamine

	**5-aminovaleric acid betaine (5AVAB)**	**(peripheral) serotonin (5HT)**	**1-methylhistamin (1MH)**
**Biosynthesis: origin and contributing enzymes**	microbiota-derived<br>DavB, DavA (out of trimethyllysine)[9,10]<br><br>breakdown product of proteins[9]	enterochromaffin cells (95%)[11,12]<br>TDC, CYP71D1 (out of tryptophan)[13]<br><br>microbiota-derived[9]	HDC, HNMT (out of histidine)[13]
**Storage compartment**	metabolically active tissue (hearth, muscle, brown fat tissue)[9]	platelets[11,12]	unknown
**Metabolism**	unknown	MAO, ALDH → 5-HIAA[13] (enterocytes, liver, platelets, kidney)<br>SNAT, ASMT → melatonin[13]<br>TAMT → N-methyl-5HT[13]<br>IDO → formyl-5-hydroxykynurenamine[13]<br>SMT → 5-methoxytryptamine[13]	MAO, ALDH → methylimidazoleacetic acid[13]
**Transport**	substrate: OCT2 & MATE1, OATP1B1 (minor uptake), OATP1B3 (minor uptake)[14]<br><br>no substrate: OAT1, OAT3, P-gp[14]<br><br>inhibition of OCTN2[15]	substrate: OCT2 & MATE1[14], OCT1[16], SERT[11], OAT3 (minor uptake)[14]<br><br>no substrate: OAT1, OATP1B1, OATP1B3, P-gp[14], OAT2, OAT4[17]	substrate: OCT2 & MATE1, P-gp (minor transport)[14]<br><br>no substrate: OAT1, OAT3, OATP1B1, OATP1B3[14]
**Dietary sources**	milk, meat, precursor in whole-grain products[9,10]	fruits, vegetables[9]	↑ in urine after intake of histamine-rich meals[18]

DavB: lysine monooxygenase, DavA: 5-aminovaleramid hydroxylase, TDC: tryptophan decarboxylase, CYP71D1: tryptamine 5-hydroxylase, MAO: monoamine oxidase, ALDH: aldehyde dehydrogenase, 5-HIAA: 5-hydroxyindoleacetate, SNAT: 5HT N-acetyltransferase, ASMT: Acetyl-5HT O-methyltransferase, TAMT: tryptamine N-methyltransferase, IDO: indoleamine 2,3-dioxygenase, SMT: 5HT methyltransferase,
OCT: organic cation transporter, MATE: multidrug and toxin extrusion protein, OAPT: organic anion transporting polypeptide, OAT: organic anion transporter, P-gp: P-glycoprotein, OCTN2: organic cation / carnitine transporter 2, SERT: 5HT transporter, HDC: histidine decarboxylase, HNMT: histamine N-methyltransferase

Table S6: Strengths and weaknesses of metformin, N^1-methylnicotinamide, creatinine, 5-aminovaleric acid betaine, serotonin and 1-methylhistamine for assessment of OCT2/MATE-mediated drug-drug interactions

	Strengths	Weaknesses
**Metformin**	- high data availability<br>- no metabolism[19]<br>- high contribution of tubular secretion (76 %)[20]<br>- regulatory recommendation[21]	- known involvement of other transporters on the disposition (OCT1, OCT3, PMAT)[22,23]<br>- metformin DDI complex and rather small in magnitude[20,24]<br>- systemic pharmacokinetics does not necessarily predict pharmacodynamic effects[24]
**N^1-Methylnicotin-amide (NMN)**	- high data availability<br>- correlation with metformin assessed with several OCT2/MATE inhibitors[4,25–28]<br>- high contribution (70%) of tubular secretion to Cl_R[26]	- circadian rhythm[4,29]<br>- special pharmacokinetics (reduction of plasma C_{max} due to several OCT2/MATE inhibitors)[4,28,29]<br>- potential impact of metabolism[30]
**Creatinine**	- high data availability<br>- correlation with metformin assessed with several OCT2/MATE inhibitors[25–28]<br>- cost-effective and reliable quantification methods[31]<br>- in vitro transport data for several transporters[32]	- relatively low (30 %) contribution of tubular secretion to Cl_R[33]<br>- known involvement of other renal transporters (OAT2)[34]<br>- dependency of plasma concentrations on muscle mass and diet[35]
**5-Aminovaleric acid betaine (5AVAB)**	- pronounced reduction of Cl_R by trimethoprim (93.6%)<br>- consistent data with cimetidine and trimethoprim[36]<br>- in vitro characterization as OCT2/MATE1 substrates and evaluation of specificity[14]	- potential impact of diet<br>- special pharmacokinetics (reduction of plasma concentrations due to cimetidine and trimethoprim)<br>- biosynthesis and metabolism in humans largely unknown<br>- contribution of renal filtration, secretion and reabsorption to Cl_R unknown
**Serotonin (5HT)**	- pronounced reduction of Cl_R by trimethoprim (73.8 %)<br>- consistent data with cimetidine and trimethoprim[36]<br>- in vitro characterization as OCT2/MATE1 substrates and evaluation of specificity[14]	- potential impact of diet<br>- several enzymes involved in biosynthesis and metabolism[13]<br>- contribution of renal filtration, secretion and reabsorption to Cl_R unknown
**1-Methylhistamine (1MH)**	- pronounced reduction of $Ae_{0\text{-}24h}$ by trimethoprim (51.9%)<br>- consistent data with cimetidine and trimethoprim[36]<br>- in vitro characterization as OCT2/MATE1 substrates and evaluation of specificity[14]	- potential impact of diet<br>- dependency on histamine levels (e.g., anaphylactic reactions)[37]<br>- analytically challenging in plasma samples due to low concentration<br>- several enzymes involved in biosynthesis and metabolism[13]

OCT: organic cation transporter, MATE: multidrug and toxin extrusion protein, DDI: drug-drug interaction, PMAT: plasma monoamine transporter, OAT2: organic anion transporter 2, Cl_R: renal clearance, $Ae_{0\text{-}24h}$: amount excreted into urine over 24 h

Figure S1

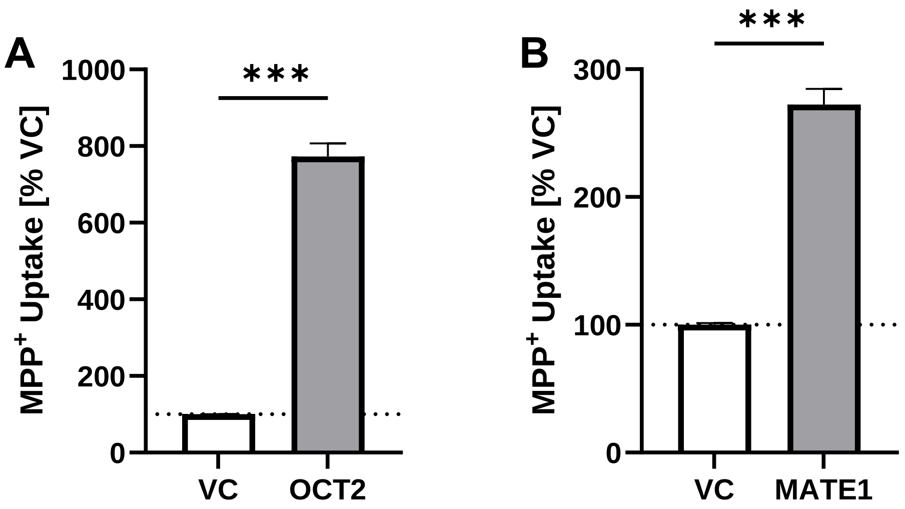

Intracellular accumulation of radiolabelled 1-methyl-4-phenylpyridinium (MPP^+, 10 µM) in HEK cells stably overexpressing (A) OCT2 or (B) MATE1 and in the respective control cells (VC). Intracellular accumulation in VC cells was set to 100% and is represented by the dashed line. Positive controls were performed for each inhibition experiment with three biological replicates per experimental setup. Data are presented as mean ± SEM. Statistical significance was analysed with a two-tailed unpaired Student's t–test in GraphPad Prism 10.6.1 (GraphPad Software; San Diego, USA). *** $p < 0.001$

Figure S2

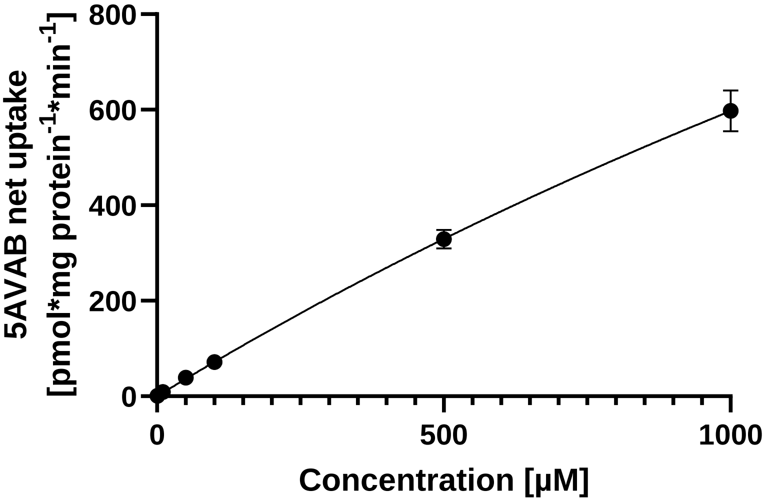

Concentration dependent uptake of 5-aminovaleric acid betaine (5AVAB) in HEK-OCT2 cells at increasing substrate concentrations and an incubation time of 5 min. Data are presented as net uptake in pmol*mg protein^{-1}*min^{-1} and are shown as mean ± SEM resulting of six biological replicates. Net uptake was calculated by subtracting values found in HEK-VC cells from values found in HEK-OCT2 cells. In case of small SEM, error bars are partially hidden behind the data point symbols.

Figure S3

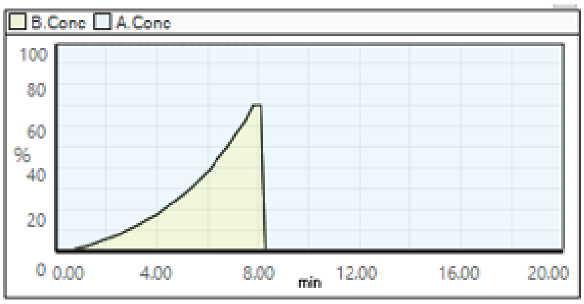

Gradient program of the method used for the quantification of 5-aminovaleric acid betaine, serotonin and 1-methylhistamine in plasma and urine samples. Eluent A consisted of 10 mmol/L ammonium formate in a mixture of acetonitrile and water (95:5) with 0.1% formic acid, whereas eluent B was 10 mmol/L ammonium formate in water with 0.1% formic acid. The total run time was 20 min. The graphical representation of the gradient program was taken from the Sciex OS 4.0 software (AB Sciex Ptc. Ltd.; Darmstadt, Germany).

SUPPLEMENTARY REFERNCES

1. InfectoTrimet® Tabletten. InfectoPharm. Accessed June 5, 2026. https://www.infectopharm.de/praeparate/infectotrimet-tabletten/

2. Gleckman R, Blagg N, Joubert DW. Trimethoprim: Mechanisms of action, antimicrobial activity, bacterial resistance, pharmacokinetics, adverse reactions, and therapeutic indications. *Pharmacotherapy: The Journal of Human Pharmacology and Drug Therapy*. 1981;1(1):14-19. doi:10.1002/j.1875-9114.1981.tb03548.x

3. Friesen WT, Hekster YA, Vree TB. Trimethoprim: Clinical use and pharmacokinetics. *Drug Intelligence & Clinical Pharmacy*. 1981;15(5):325-330. doi:10.1177/106002808101500502

4. Müller F, Pontones CA, Renner B, et al. N^1-Methylnicotinamide as an endogenous probe for drug interactions by renal cation transporters: studies on the metformin–trimethoprim interaction. *Eur J Clin Pharmacol*. 2015;71(1):85-94. doi:10.1007/s00228-014-1770-2

5. Grün B, Kiessling MK, Burhenne J, et al. Trimethoprim-metformin interaction and its genetic modulation by OCT2 and MATE1 transporters. *Br J Clin Pharmacol*. 2013;76(5):787-796. doi:10.1111/bcp.12079

6. Kosoglou T, Rocci ML, Vlasses PH. Trimethoprim alters the disposition of procainamide and N-acetylprocainamide. *Clin Pharmacol Ther*. 1988;44(4):467-477. doi:10.1038/clpt.1988.181

7. Moore KHP, Yuen GJ, Raasch RH, et al. Pharmacokinetics of lamivudine administered alone and with trimethoprim-sulfamethoxazole. *Clinical Pharmacology & Therapeutics*. 1996;59(5):550-558. doi:10.1016/S0009-9236(96)90183-6

8. Lee BL, Safrin S, Makrides V, Gambertoglio JG. Zidovudine, trimethoprim, and dapsone pharmacokinetic interactions in patients with human immunodeficiency virus infection. *Antimicrob Agents Chemother*. 1996;40(5):1231-1236. doi:10.1128/AAC.40.5.1231

9. Wishart DS, Guo A, Oler E, et al. HMDB 5.0: The Human Metabolome Database for 2022. *Nucleic Acids Res*. 2022;50(D1):D622-D631. doi:10.1093/nar/gkab1062

10. Haikonen R, Kärkkäinen O, Koistinen V, Hanhineva K. Diet- and microbiota-related metabolite, 5-aminovaleric acid betaine (5-AVAB), in health and disease. *Trends Endocrinol Metab*. 2022;33(7):463-480. doi:10.1016/j.tem.2022.04.004

11. Yabut JM, Crane JD, Green AE, Keating DJ, Khan WI, Steinberg GR. Emerging roles for serotonin in regulating metabolism: New implications for an ancient molecule. *Endocr Rev*. 2019;40(4):1092-1107. doi:10.1210/er.2018-00283

12. Jonnakuty C, Gragnoli C. What do we know about serotonin? *J Cell Physiol*. 2008;217(2):301-306. doi:10.1002/jcp.21533

13. Kanehisa M, Furumichi M, Sato Y, Kawashima M, Ishiguro-Watanabe M. KEGG for taxonomy-based analysis of pathways and genomes. *Nucleic Acids Res*. 2023;51(D1):D587-D592. doi:10.1093/nar/gkac963

14. Gessner A, Picurová J, Englhard L, Müller F, Fromm MF, König J. Putative new biomarkers for renal transporter-mediated drug-drug interactions: Characterization as

substrates of organic cation transporter 2, multidrug and toxin extrusion protein 1, and other important drug transporters. *Drug Metab Dispos*. 2025;53(10):100155. doi:10.1016/j.dmd.2025.100155

15. Kärkkäinen O, Tuomainen T, Koistinen V, et al. Whole grain intake associated molecule 5-aminovaleric acid betaine decreases β-oxidation of fatty acids in mouse cardiomyocytes. *Sci Rep*. 2018;8(1):13036. doi:10.1038/s41598-018-31484-5

16. Boxberger KH, Hagenbuch B, Lampe JN. Common drugs inhibit human organic cation transporter 1 (OCT1)-mediated neurotransmitter uptake. *Drug Metab Dispos*. 2014;42(6):990-995. doi:10.1124/dmd.113.055095

17. Ma Y, Wang X, Gou X, Wu X. Identification and characterization of an endogenous biomarker of the renal vectorial transport (OCT2-MATE1). *Biopharm & Drug Disp*. 2024;45(1):43-57. doi:10.1002/bdd.2382

18. Hermann K, Hertenberger B, Ring J. Measurement and characterization of histamine and methylhistamine in human urine under histamine-rich and histamine-poor diets. *Int Arch Allergy Immunol*. 1993;101(1):13-19. doi:10.1159/000236493

19. Gong L, Goswami S, Giacomini KM, Altman RB, Klein TE. Metformin pathways: pharmacokinetics and pharmacodynamics. *Pharmacogenet Genomics*. 2012;22(11):820-827. doi:10.1097/FPC.0b013e3283559b22

20. Krishnan S, Ramsden D, Ferguson D, et al. Challenges and opportunities for improved drug–drug interaction predictions for renal OCT2 and MATE1/2-K transporters. *Clinical Pharmacology & Therapeutics*. 2022;112(3):562-572. doi:10.1002/cpt.2666

21. European Medicines Agency (EMA). ICH M12 Guideline on drug interaction studies. Published online May 21, 2024. Accessed September 24, 2024. https://www.ema.europa.eu/en/documents/scientific-guideline/ich-m12-guideline-drug-interaction-studies-step-5_en.pdf

22. Zhou M, Xia L, Wang J. Metformin transport by a newly cloned proton-stimulated organic cation transporter (plasma membrane monoamine transporter) expressed in human intestine. *Drug Metab Dispos*. 2007;35(10):1956-1962. doi:10.1124/dmd.107.015495

23. Graham GG, Punt J, Arora M, et al. Clinical pharmacokinetics of metformin. *Clin Pharmacokinet*. 2011;50(2):81-98. doi:10.2165/11534750-000000000-00000

24. Zamek-Gliszczynski MJ, Chu X, Cook JA, et al. ITC commentary on metformin clinical drug-drug interaction study design that enables an efficacy- and safety-based dose adjustment decision. *Clinical Pharmacology & Therapeutics*. 2018;104(5):781-784. doi:10.1002/cpt.1082

25. Nishii R, Xue Y, Huo R, et al. Evaluating the utility of endogenous OCT2 and MATE1/2-K biomarkers for DDI assessment in early clinical settings. *J Pharm Sci*. 2025;114(12):103776. doi:10.1016/j.xphs.2025.103776

26. Miyake T, Kimoto E, Luo L, et al. Identification of appropriate endogenous biomarker for risk assessment of multidrug and toxin extrusion protein-mediated drug-drug interactions

in healthy volunteers. *Clinical Pharmacology & Therapeutics*. 2021;109(2):507-516. doi:10.1002/cpt.2022

27. Koishikawa T, Fujiwara K, Taskar K, et al. Effects of cimetidine and dolutegravir on the endogenous drug-drug interaction biomarkers for organic cation transporter 2 and multidrug and toxin extrusion protein 1 in healthy volunteers. *Clin Pharmacol Ther*. 2025;117(2):523-533. doi:10.1002/cpt.3482

28. Müller F, Hohl K, Keller S, et al. N^1-Methylnicotinamide as biomarker for MATE-mediated renal drug–drug interactions: Impact of cimetidine, rifampin, verapamil, and probenecid. *Clin Pharmacol Ther*. 2023;113(5):1070-1079. doi:10.1002/cpt.2849

29. Ito S, Kusuhara H, Kumagai Y, et al. *N*-Methylnicotinamide is an endogenous probe for evaluation of drug-drug interactions involving multidrug and toxin extrusions (MATE1 and MATE2-K). *Clin Pharmacol Ther*. 2012;92(5):635-641. doi:10.1038/clpt.2012.138

30. Kitamura S, Nitta K, Tayama Y, et al. Aldehyde oxidase-catalyzed metabolism of *N*1-methylnicotinamide in vivo and in vitro in chimeric mice with humanized liver. *Drug Metabolism and Disposition*. 2008;36(7):1202-1205. doi:10.1124/dmd.107.019075

31. Pottel H, Delanaye P, Cavalier E. Exploring renal function assessment: Creatinine, cystatin C, and estimated glomerular filtration rate focused on the European Kidney Function Consortium equation. *Ann Lab Med*. 2024;44(2):135-143. doi:10.3343/alm.2023.0237

32. Ma Y, Zhang M, Yang J, Zhu L, Dai J, Wu X. Characterization of the renal tubular transport of creatinine by activity-based protein profiling and transport kinetics. *European Journal of Pharmaceutical Sciences*. 2023;180:106342. doi:10.1016/j.ejps.2022.106342

33. Mathialagan S, Feng B, Rodrigues AD, Varma MVS. Drug-drug interactions involving renal OCT2/MATE transporters: Clinical risk assessment may require endogenous biomarker-informed approach. *Clin Pharmacol Ther*. 2021;110(4):855-859. doi:10.1002/cpt.2089

34. Shen H, Liu T, Morse BL, et al. Characterization of organic anion transporter 2 (SLC22A7): A highly efficient transporter for creatinine and species-dependent renal tubular expression. *Drug Metabolism and Disposition*. 2015;43(7):984-993. doi:10.1124/dmd.114.062364

35. Kashani K, Rosner MH, Ostermann M. Creatinine: From physiology to clinical application. *European Journal of Internal Medicine*. 2020;72:9-14. doi:10.1016/j.ejim.2019.10.025

36. Gessner A, König J, Wenisch P, et al. New biomarkers for renal transporter-mediated drug–drug interactions: Metabolomic effects of cimetidine, probenecid, verapamil, and rifampin in humans. *Clin Pharmacol Ther*. 2024;117(1):130-142. doi:10.1002/cpt.3414

37. Keyzer JJ, Breukelman H, Wolthers BG, Richardson FJ, de Monchy JG. Measurement of N^{τ}-methylhistamine concentrations in plasma and urine as a parameter for histamine release during anaphylactoid reactions. *Agents Actions*. 1985;16(3-4):76-79. doi:10.1007/BF01983105
